# Informed consent and Italian physicians: change course or abandon ship—from formal authorization to a culture of sharing

**DOI:** 10.1007/s11019-015-9637-6

**Published:** 2015-04-05

**Authors:** Emanuela Turillazzi, Margherita Neri

**Affiliations:** University of Foggia, Foggia, Italy

**Keywords:** Informed consent, Consent forms, Defensive medicine, Medical malpractice, Health system quality

## Abstract

In Italy in recent years, an exponential increase in the frequency of medical malpractice claims relating to the issue of informed consent has substantially altered not only medical ethics, but medical practice as well. Total or partial lack of consent has become the cornerstone of many malpractice lawsuits, and continues to be one of the primary cudgels against defendant physicians in Italian courtrooms. Physicians have responded to the rising number of claims with an increase in ‘defensive medicine’ and a prevailing preoccupation with the purely formal aspects of consent. The result is a plethora of consent forms, believed to be a guarantee of ‘informed consent’, as well as a growing reliance on informed consent as a shield against judicial action brought by the patient. Physicians ‘inform’ patients without really sharing information, often delegating the task of communication to other professionals who are not doctors. Italian judges always condemn the physician when information to the patient has been inadequate, thus leading insurance companies to consider the lack of valid informed consent as the total responsibility of the physician and/or the hospital. It is necessary to change tack, to remove this idea of consent which permeates the defensive culture of medical practice. Italian physicians need to be trained, first of all, to become aware that information and consent are two distinct processes, albeit closely connected. Valid communication (in which there is information and consent) demands a higher level of professionalism from physicians.

## Introduction

In Italy in recent years, an exponential increase in the frequency of medical malpractice claims relating to the issue of informed consent has substantially altered not only medical ethics, but medical practice as well (Traina 2009; Elli et al. 2013). Total or partial lack of consent has become the cornerstone of many malpractice lawsuits, and continues to be one of the primary cudgels against defendant physicians in Italian courtrooms. Physicians have responded to the rising number of claims with an increase in ‘defensive medicine’ and a prevailing preoccupation with the purely formal aspects of consent. The result is a plethora of consent forms, believed to be a guarantee of ‘informed consent’, as well as a growing reliance on informed consent as a shield against judicial action brought by the patient. Informed consent is, then, an important matter in our currently litigious society. The conceptions that emerge from the current scenario are problematic in a number of ways. If we think of consent in this way, we are in danger of forgetting that it is a process, and not merely a slip of paper, and it thus requires attention to a number of significant ethical issues.

## What is informed consent?

Medical ethicists generally hold that for consent to be effective it needs to be informed. It is worth drawing attention to the ambiguity of the phrase ‘informed consent’, the use of which has oriented the attention of health professionals towards the formalities of the question. Yet as far back as 1992, the Italian National Bioethics Committee, in a fundamental document on ‘Information and consent to medical intervention’, underlined the distinction between the two aspects, the former (‘information’) being an indispensable and fundamental requirement of the latter (‘consent’) (Italian National Bioethics Committee 1992).

Informed consent to medical intervention is not necessarily synonymous with conscious agreement. The two processes, one informative and one the expression of consent, are indiscernible one from the other, but should be dealt with separately and specifically. Consent to medical intervention infers a more profound relationship between patient and physician (Halpern 2014), which cannot be encapsulated in a signature on an acceptance form. The phrase ‘consent consciously given’ would thus seem more appropriate since ‘informed’ does not describe the consent, but the patient who gives it.

That Italy is slow to develop a true culture of consent is clear from the profusion of consent forms in existence today. Far from fulfilling an efficient communicative function, which by nature is alien to medical bureaucracy and officialdom, these forms give communication an ambiguous twist. The complexity of informed consent cannot be reduced to three words, ‘please sign here’ (Shokrollahi 2010). The answer to the question ‘what is informed consent?’ can only be: ‘one thing it is not, and that is a form that the patient signs’ (Jerrold 2011, p 133). Physicians don’t give informed consent, they get it. What they give is information (Jerrold 2011, p. 133). The perfunctory, *pro forma* signing of a consent form elicits mere passive assent, not active consent. It neither enhances the patient’s understanding nor helps him or her to take responsibility for the choices made.

## Italian deontological codes

The Italian deontological codes have shown an increasing awareness of the importance of ‘consent’ as the final act in the communicative process. Since 1970 in Italy, successive codes have expressed a deep change in the physician–patient relationship. In particular, the topic of informed consent has assumed a central place in deontological codes. Once essential only when there was a risk for the patient, now it is a necessary concern for the physician. Since consent is just a part of the decision-making process, it has to be accomplished through the complete and comprehensible disclosure of all information regarding treatment, options and possible consequences. Attention should not be focused only on consent; the Italian code of medical deontology awards considerable importance to the central aspect of information (Fineschi et al. 1997; Sacchini and Antico 2000; Surbone et al. 2004). The latest code (2014) gives a major role to ‘communicative relation’ as an indispensable condition to allow health workers to give, and patients to receive, all the necessary information for the latter to reach a conscious decision regarding the diagnostic-therapeutic options open to them. Therefore it appears that Italian deontological sensibility now embraces and accepts the spirit of consent which expresses the essential and central nature of patient autonomy.

## The paradox of informed consent

Various factors have favored the distorted behavior of Italian physicians in relation to information and consent. Their attitudes have been prompted and conditioned firstly by an explosion of lawsuits against physicians, based on presumed lack of information and absence of consent and secondly, by their consequent attempt to stem this phenomenon. ‘Informed consent’ has thus been formalized and, often at the behest of insurance companies, transformed into a signature on a consent form. Thirdly, fear of judicial consequences affects medical procedures, causing physicians to orient their professional conduct according to the law to shield themselves from possible legal action.

This conditioning has put a strain on physician–patient relations and has fed into a process which, paradoxically, has induced physicians to shy away from interpersonal relationships with the patient. Consent to medical intervention has become a real bogeyman for health workers and hospitals and, consequently, for the insurance companies which cover the medical responsibility of both, since it constitutes a growing economic risk. Physicians increasingly attribute legal significance to informed consent, placing it more highly than ethical values. They ‘inform’ patients without really sharing information, often delegating the task of communication to other professionals who are not doctors. Italian judges always condemn the physician when information to the patient has been inadequate, thus leading insurance companies to consider the lack of valid informed consent as the total responsibility of the physician and/or the hospital. Most health risk policies offered by insurance companies extend no or limited coverage in the absence of informed consent.

In Italy we are now witnessing a shifting of the parameters of consent: from a central place in the physician–patient relationship to an unpleasant task for the physician, achieved through the mere signing of a form, usually for their own protection. From a way to satisfy a shared interest in obtaining the best possible results from treatment, it has become a highly controversial legal issue.

Looking at the Italian Courts, we found more and more increasing emphasis on information. So the Italian jurisprudence widely cleared that the duty to provide information concerns the intervention, its unavoidable difficulties, the attainable effects and the possible risks, so as to place the patient in conditions to decide on the opportunity to proceed to it or to omit it through the balance of advantages and risks. Over the years, there has been an increasing expansion of the boundaries of information “The physician must provide, in a complete and comprehensive way, all the scientifically validate information about the proposed treatment, and illustrate the effects, the risks of failure, and any adverse event. Also the anomalous or unlikely risks and adverse events—if known to medical science and not completely abnormal—must be communicated, so that the patient can consciously decide whether to take the risks of treatment or endure the disease, especially in cases where the medical or surgical treatment is not essential for the patient’s survival”. Judges come, therefore, to affirm that changes in the operating room schedule (e.g. laparotomic conversion, viscerolisis and bilateral ovarian resection in a case of laparoendoscopic approach for fertility management) if non-urgent and necessary to save the life of the patient cannot be performed without obtaining the consent of the patient. The consensus is so imperative that it is not relevant that the surgical intervention was technically correct, for the simple reason that because of total lack of information, the patient was not able to assent to the change in the surgery’s plan. In this case an injury to his/her personal dignity was made in a crucial moment of his/her life. Judicial decisions have subtly expanded the doctrine of informed consent beyond its traditional limits, thus defining an obligation to provide information which does not concern only the risks related to a particular diagnostic and/or therapeutic performance, but also the real, maybe temporary lacking situation of the hospital, with respect to the supplies and the equipments and their regular functioning, so that the patient can decide not only whether to undertake the intervention or not, but also whether to do it in that hospital or to ask to be transferred to a better and more adequately equipped one. The leading case is represented by a case of a newborn affected by cerebral palsy, in which the physician has been condemned to answer together with the hospital for compensation for damage, because he did not inform the patient, pregnant, about the temporary absence in the hospital in which he operated of a working cardiotocograph to monitor the foetus condition: “…the physician must inform the patients about the inadequacies of such equipments in the hospital, also in case of their temporary unavailability, and about the greater risk involved for the safety of the procedure because of the lack of a specific diagnostic instrument…”.

Undoubtedly, a great contribution to the diffusion of informed consent in Italy came from the judges’ pronouncements, who greatly influenced the evolution of medical deontology. Whilst the Italian Courts seem promote a sort of “contexualized informed consent” whereby the physicians provide information tailored to the patients and respect patient autonomy (Wells and Kaptchuk 2012), the real risk is that of a sort of bureaucratization of informed consent.

## Abandon the ship of this ‘consent’?

Bluntly stated, this model of informed consent as the formal authorization of medical intervention in the (illusory) hope of limiting judicial litigation has failed. It has undermined the spirit of interpersonal physician–patient relations based on effective communication. This informed consent wastes money and time and offers no benefit, either to physicians or to patients. The time has come to abandon ship; to forego a model in which consent has become a ‘conduit/container’ (Manson and O’Neill 2007; Bullock 2010) for disclosure for the patient’s decision-making and the physician’s legal protection. In other words, and unfortunately, those who seek consent (the physicians) are concerned only with formalizing (by a signature on the consent form) the flow of information to those who have to decide whether to consent or not (the patients).

In this perspective, the use of encyclopedic ‘forms’ listing all the possible risks and complications, acts as a shield against legal action should the physician fail to fully explain the likely outcome and all possible negative and adverse events. However, to burden the patient with every possible risk of an operation may not be in his or her interests (Kocarnik 2014). Over-zealous warnings of the risks involved is another form of defensive medicine in which protection of the surgeon against litigation can easily become too strong a motive.

The fact is that while Italian physicians do not really subscribe to a model of communicative relations, informed consent continues to be used to protect them from lawsuits. This reveals the physicians’ unrequited hope that if things somehow go wrong, a signature on a consent form will magically confer protection against a lawsuit or jury verdict. In reality, this is not true, as has finally been pointed out by Italian judges: “The physician fails to fulfil his obligation to supply valid and exhaustive informed consent to the patient not only when he or she completely omits to describe the medical procedures, the risks and possibilities of success, but also when the same physician gives a generalized form to the patient to sign, from which it is not possible to deduce with certainty that the patient has obtained all the information he or she needs”.

Nevertheless, Italian physicians generally continue to believe that communications begin and end with the signature on a consent form, just as they believe in its ability to protect them.

It is necessary to change tack, to remove this idea of consent which permeates the official and defensive culture of medical practice. Italian physicians need to be trained, first of all, to become aware that information and consent are two distinct processes, albeit closely connected. Valid communication (in which there is information and consent) demands a higher level of professionalism from physicians, however communication skills and abilities have only in the past couple decades received focused attention in the training of health care professionals (Makoul 2003).

## Conclusions

In Italy, a gradual transformation has been underway now for several years. The relationship of trust between citizens and the national health service is strengthening through a long process whose key points are the efficiency of the health system, the appropriateness of services, and the accountability of all health professionals. There is a risk, however, that all the strategies and measures which support this process will not achieve their goal if they fail to take account of the central issue in building relations of trust with citizen-patients. Patients have a right to information, and thus the right to depend upon a health organization which not only guarantees appropriate and safe medical care, but also respects the citizen’s rights (informed consent, the patient’s wishes, living wills, error disclosure etc.).

An excessive use of formalities, fear of being sued and defensive medicine orient the professional behaviour of Italian physicians more towards preventing sanctions rather than towards good practice (Di Landro 2012; Genovese et al. 2014). Bioethical considerations and the inspiring principles of medical deontology accentuate other inspiring principles of the medical profession in the building of that close relationship between physician/hospital and citizen/patient which is fundamental for the growth of any health system. Therapeutic alliance, medical intervention and clinical and organizational appropriateness should have as their common denominator, informed consent. The consent of the patient founded on correct (ways and times) and adequate information supplied by the physician (and the staff) in a suitable context, is the preliminary condition for a health service which is able to guarantee the patient’s right to health as well as the right to decide how to be treated. The process of informed consent could and should also be the pivotal point around which the physician exercises the right to practice his/her profession to the full.

Communicating with patients is considered to be central to the clinical abilities of health professionals world-wide. To protect the health of patients, and contrary to the current tendency to over-prescribe and carry out an excessive and unjustifiable number of useless tests, we need to overcome our ‘fear’ of informed consent. In fact, good communication skills have been linked not only to greater patient adherence to treatment, better patient health outcomes, reduced patient anxiety, increased recall, and improved understanding, but also to fewer physician malpractice claims. More emphasis on the core of communication skills is needed and training them is essential in improving doctor–patient communication skills.

Research has revealed that professional communication can be acquired though communication skills training in medical education (Makoul 2003). Communication skills program should be more widely integrated into medical education and training program since doctor–patient communication is a key element in teaching at all levels, including undergraduate and postgraduate medical programs, residency training, and continuing medical education. Moreover, educational intervention and medical staff training on doctor–patient communication in hospitals are increasingly advocated and should be incorporated into training for healthcare providers since they are associated with improvements in malpractice prevention and risk-management (Catino and Celotti 2009; Turillazzi and Neri 2014).

While they may sometimes have conflicting interests, physicians, patients, hospital and managed care executives can work together to restore meaning to the ethical and legal concept of informed consent, especially now that the latter is taking on greater prominence in medical malpractice litigation.

## References

[CR1] Bullock E (2010). Informed consent as waiver: The doctrine rethought?. Ethical Perspectives.

[CR2] Catino M, Celotti S (2009). The problem of defensive medicine: Two Italian surveys. Studies in Health Technology and Informatics.

[CR3] Di Landro AR (2012). Criminal law as a response to medical malpractice: Pluses and minuses–comparing Italian and US experiences. Medicine and Law.

[CR4] Elli L (2013). Defensive medicine practices among gastroenterologists in Lombardy: Between lawsuits and the economic crisis. Digestive and Liver Disease.

[CR5] Fineschi V (1997). The new Italian code of medical ethics. Journal of Medical Ethics.

[CR6] Genovese U (2014). Defensive medicine and outcomes for medical malpractice liability. Igiene e Sanità Pubblica.

[CR7] Halpern J (2014). From idealized clinical empathy to empathic communication in medical care. Medicine, Health Care and Philosophy.

[CR8] Italian National Bioethics Committee. Information and consent related to medical acts. Rome, 20 June 1992. http://www.governo.it/bioetica/ Accessed 20 Dec 2014.

[CR9] Jerrold L (2011). Litigation and legislation: What do patients actually consent to?. American Journal of Orthodontics and Dentofacial Orthopedics.

[CR10] Kocarnik JM (2014). Disclosing controversial risk in informed consent: How serious is serious?. American Journal of Bioethics.

[CR11] Makoul G (2003). Communication skills education in medical school and beyond. JAMA.

[CR12] Manson N, O’Neill O (2007). Rethinking informed consent in bioethics.

[CR13] Sacchini D, Antico L (2000). The professional autonomy of the medical doctor in Italy. Theoretical Medicine and Bioethics.

[CR14] Shokrollahi K (2010). Request for treatment: The evolution of consent. Annals of the Royal College of Surgeons of England.

[CR15] Surbone A (2004). Evolution of truth-telling attitudes and practices in Italy. Critical Reviews in Oncology/Hematology.

[CR16] Traina F (2009). Medical malpractice: The experience in Italy. Clinical Orthopaedics and Related Research.

[CR17] Turillazzi E, Neri M (2014). Medical error disclosure: from the therapeutic alliance to risk management: the vision of the new Italian code of medical ethics. BMC Medical Ethics.

[CR18] Wells RE, Kaptchuk TJ (2012). To tell the truth, the whole truth, may do patients harm: The problem of the nocebo effect for informed consent. American Journal of Bioethics.

